# Supplementary material for: Effects of intraocular pressure change on intraocular lens power calculation in primary open-angle glaucoma and ocular hypertension
Source: PLoS One. 2024 Jun 10;19(6):e0304169. doi: 10.1371/journal.pone.0304169 (PMC11164340; doi:10.1371/journal.pone.0304169)
Supplement: S1 Table — OHT ocular hypertension, POAG primary open angle glaucoma, IOP Pre intraocular pressure before IOP reduction, IOP Post intraocular pressure after IOP reduction, IOL Power Pre intraocular lens power before IOP reduction, IOL Power Post intraocular lens power after IOP reduction, AL Pre axial length before IOP reduction, AL Post axial length after IOP reduction, ACD Pre anterior chamber depth before IOP reduction, ACD Post anterior chamber depth after IOP reduction, CCT Pre central corneal thickness before IOP reduction, CCT Post central corneal thickness after IOP reduction, K1 Pre flattest keratometry before IOP reduction, K1 Post flattest keratometry after IOP reduction, K2 Pre steepest keratometry before IOP reduction, K2 Post steepest keratometry after IOP reduction, D diopters. (DOCX) [file pone.0304169.s001.docx]

| **Eye no.** | **Sex** | **Age (year)** | **Diagnosis** | **IOP  Pre (mmHg)** | **IOP Post (mmHg)** | **IOL Power Pre (D)** | **IOL Power Post (D)** | **AL  Pre (mm)** | **AL  Post (mm)** | **ACD  Pre (mm)** | **ACD  Post (mm)** | **CCT  Pre (µm)** | **CCT  Post (µm)** | **K1 Pre (D)** | **K1 Post (D)** | **K2 pre (D)** | **K2**  **Post (D)** |
| --- | --- | --- | --- | --- | --- | --- | --- | --- | --- | --- | --- | --- | --- | --- | --- | --- | --- |
| 1 | male | 50 | OHT | 26 | 17 | 21.5 | 21.5 | 24.6 | 24.01 | 3 | 2.87 | 599 | 597 | 42.08 | 41.93 | 42.19 | 42.08 |
| 2 | male | 50 | OHT | 26 | 17 | 21.5 | 21.5 | 23.95 | 23.93 | 2.96 | 2.9 | 598 | 607 | 41.87 | 41.87 | 42.72 | 42.51 |
| 3 | female | 52 | OHT | 25 | 19 | 20.5 | 20.5 | 22.97 | 22.96 | 3.3 | 3.06 | 572 | 572 | 45.98 | 45.86 | 46.55 | 46.42 |
| 4 | female | 52 | OHT | 27 | 18 | 21 | 21 | 22.68 | 22.69 | 3.34 | 3.33 | 574 | 574 | 46.3 | 46.17 | 47.27 | 47.07 |
| 5 | male | 54 | POAG | 25 | 18 | 13 | 12 | 25.79 | 25.77 | 3.91 | 3.98 | 522 | 514 | 44.58 | 45.18 | 44.94 | 46.04 |
| 6 | male | 54 | POAG | 25 | 16 | 19 | 19 | 24.02 | 24.01 | 3.76 | 3.75 | 540 | 531 | 44.23 | 44.35 | 44.88 | 44.94 |
| 7 | female | 59 | POAG | 30 | 20 | 24.5 | 24.5 | 22.27 | 22.21 | 2.87 | 2.89 | 559 | 527 | 44.23 | 44.41 | 44.94 | 44.82 |
| 8 | female | 60 | OHT | 25 | 19 | 23 | 23 | 22.56 | 22.5 | 3.63 | 3.53 | 558 | 563 | 45 | 45.24 | 45.42 | 45.55 |
| 9 | female | 60 | OHT | 25 | 17 | 23 | 23 | 22.53 | 22.5 | 3.47 | 3.5 | 556 | 557 | 44.7 | 45 | 45 | 45 |
| 10 | male | 65 | POAG | 34 | 11 | 20 | 20 | 24.35 | 24.24 | 2.51 | 2.57 | 528 | 556 | 42.35 | 42.67 | 43.05 | 43.1 |
| 11 | male | 66 | OHT | 30 | 18 | 23.5 | 24 | 22.79 | 22.71 | 3.02 | 2.97 | 579 | 596 | 42.99 | 42.99 | 44.18 | 44.23 |
| 12 | male | 66 | OHT | 28 | 17 | 23 | 23.5 | 22.8 | 22.74 | 2.87 | 2.82 | 561 | 592 | 43.55 | 43.49 | 44.82 | 44.64 |
| 13 | male | 67 | POAG | 32 | 18 | 15.5 | 15.5 | 25.72 | 25.67 | 3.26 | 3.25 | 570 | 586 | 45.51 | 42.56 | 42.94 | 42.72 |
| 14 | male | 67 | POAG | 34 | 19 | 17 | 17 | 25.44 | 25.38 | 3.24 | 3.42 | 579 | 606 | 42.03 | 42.08 | 42.4 | 42.56 |
| 15 | male | 68 | POAG | 26 | 16 | 21 | 21 | 22.29 | 22.26 | 3.14 | 2.88 | 540 | 535 | 47.2 | 47.54 | 48.15 | 48.49 |
| 16 | male | 68 | OHT | 34 | 17 | 21 | 21.5 | 22.52 | 23.37 | 3.46 | 3.32 | 503 | 521 | 46.94 | 47.14 | 47.34 | 47.14 |
| 17 | male | 68 | OHT | 34 | 18 | 21.5 | 22 | 22.48 | 22.34 | 3.7 | 3.61 | 498 | 518 | 46.94 | 46.55 | 46.94 | 47.27 |
| 18 | male | 70 | POAG | 30 | 12 | 21 | 23 | 22.65 | 22.52 | 3.04 | 3.02 | 491 | 500 | 46.49 | 45.49 | 46.49 | 46.17 |
| 19 | female | 75 | OHT | 29 | 20 | 21 | 21 | 22.81 | 22.82 | 2.76 | 2.76 | 542 | 542 | 45.73 | 45.73 | 46.42 | 46.42 |
| 20 | female | 75 | OHT | 26 | 20 | 21 | 21 | 22.78 | 22.77 | 2.95 | 2.87 | 543 | 550 | 45.61 | 45.92 | 46.49 | 46.81 |
| 21 | male | 75 | POAG | 26 | 17 | 21 | 21.5 | 24.04 | 23.98 | 3.15 | 4.45 | 528 | 518 | 42.03 | 41.82 | 42.83 | 42.61 |
| 22 | female | 77 | POAG | 25 | 15 | 21 | 21 | 22.86 | 22.84 | 2.51 | 2.5 | 550 | 547 | 45.73 | 45.3 | 46.62 | 46.55 |
| 23 | female | 77 | POAG | 32 | 15 | 20.5 | 21 | 22.73 | 22.69 | 2.56 | 2.56 | 545 | 540 | 46.42 | 46.17 | 47.07 | 46.68 |
| 24 | male | 86 | OHT | 32 | 20 | 24.5 | 25 | 22.9 | 22.83 | 2.14 | 2.39 | 520 | 520 | 41.31 | 40.81 | 44.12 | 43.77 |
| 25 | male | 89 | POAG | 32 | 18 | 20.5 | 21.5 | 23.72 | 23.57 | 2.62 | 2.41 | 580 | 564 | 42.56 | 42.03 | 45.12 | 45.18 |
| 26 | male | 68 | OHT | 32 | 16 | 23 | 23 | 23 | 22.94 | 2.49 | 2.42 | 514 | 518 | 43.44 | 43.44 | 44.12 | 44.18 |
| 27 | male | 61 | OHT | 26 | 17 | 17.5 | 17.5 | 24.2 | 24.21 | 3.99 | 3.99 | 573 | 571 | 44.82 | 44.76 | 45.42 | 45.3 |
| 28 | male | 61 | OHT | 26 | 16 | 18 | 18.5 | 24.21 | 24.22 | 4.18 | 4.11 | 574 | 577 | 44.23 | 44.23 | 45.06 | 44.68 |

**S1 Table :** **Raw data from all participants**

OHT ocular hypertension, POAG primary open angle glaucoma, IOP Pre intraocular pressure before IOP reduction, IOP Post intraocular pressure after IOP reduction, IOL Power Pre intraocular lens power before IOP reduction, IOL Power Post intraocular lens power after IOP reduction, AL Pre axial length before IOP reduction, AL Post axial length after IOP reduction, ACD Pre anterior chamber depth before IOP reduction, ACD Post anterior chamber depth after IOP reduction, CCT Pre central corneal thickness before IOP reduction, CCT Post central corneal thickness after IOP reduction, K1 Pre flattest keratometry before IOP reduction, K1 Post flattest keratometry after IOP reduction, K2 Pre steepest keratometry before IOP reduction, K2 Post steepest keratometry after IOP reduction, D diopters
